# An Fe_6_C Core in All Nitrogenase Cofactors

**DOI:** 10.1002/anie.202209190

**Published:** 2022-09-07

**Authors:** Laure Decamps, Derek B. Rice, Serena DeBeer

**Affiliations:** ^1^ Department of Inorganic Spectroscopy Max Planck Institute for Chemical Energy Conversion Stiftstrasse 34–36 45470 Mülheim an der Ruhr Germany

**Keywords:** Carbides, Iron-Sulfur Clusters, Nitrogenases, x-Ray Emission Spectroscopy

## Abstract

The biological process of dinitrogen reduction to ammonium occurs at the cofactors of nitrogenases, the only enzymes that catalyze this challenging chemical reaction. Three types of nitrogenases have been described, named according to the heterometal in their cofactor: molybdenum, vanadium or iron nitrogenases. Spectroscopic and structural characterization allowed the unambiguous identification of the cofactors of molybdenum and vanadium nitrogenases and revealed a central μ_6_‐carbide in both of them. Although genetic studies suggested that the cofactor of the iron nitrogenase contains a similar Fe_6_C core, this has not been experimentally demonstrated. Here we report Valence‐to‐Core X‐ray Emission Spectroscopy providing experimental evidence that this cofactor contains a carbide, thereby making the Fe_6_C core a feature of all nitrogenase cofactors.

## Graphical abstract

Earlier spectroscopic and structural studies have shown that the cofactors of molybdenum and vanadium nitrogenases contain a central μ_6_‐carbide. Here, we used valence‐to‐core X‐ray emission spectroscopy (VtC XES) to probe the presence of a carbide in the cofactor of iron nitrogenase. The presence of a Kβ′′ peak at 7100 eV reveals an interstitial carbon, showing that the cofactor of Fe nitrogenase contains an Fe_6_C core.

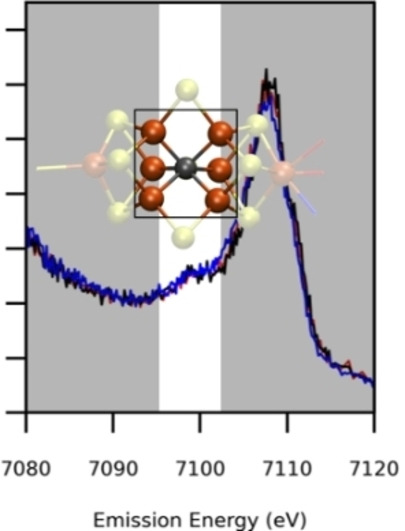

The highly energy‐demanding reduction of dinitrogen (N_2_) to ammonium (NH_4_^+^) is catalyzed by nitrogenases, enzymes produced by only a few bacteria and archaea termed diazotrophs. Three nitrogenase isozymes have been characterized and are defined based on the metal content of their cofactor: Mo, V, or Fe nitrogenase. Mo nitrogenase is the most widespread isozyme and is present in all diazotrophs identified to date, while V and Fe nitrogenases have been described as “alternative” nitrogenases, produced in Mo‐deficient conditions.^[1]^ These three homologous enzymes are genetically and biochemically distinct systems, showing different reactivities and catalytic properties. However, all three isozymes have been suggested to follow the same catalytic mechanism, and their cofactors and protein scaffolds display similar structures according to X‐ray crystallography and X‐ray absorption spectroscopy data.^[2]^ Consistently, the biosynthesis of their cofactors follows a common route, with the radical SAM enzyme NifB fusing two [4Fe:4S] cubane precursors into a [8Fe:9S:C] NifB‐co, precursor of the three cofactors: FeMoco, FeVco and FeFeco.^[3]^ The precursors of Mo and V nitrogenases are further maturated on a dedicated scaffold, namely NifEN and VnfEN.^[4]^ These scaffolds are required for the insertion of the heterometal and of the homocitrate in the cofactors. However, no such assembly scaffold has been identified for the maturation of FeFeco, although FeFeco has been suggested to contain homocitrate like FeMoco and FeVco.^[5]^ The exact composition and structure of nitrogenase cofactors remained elusive until the application of valence‐to‐core X‐ray emission spectroscopy (VtC XES), as well as an atomic resolution crystal structure, allowed the identification of the central atom of FeMoco as a carbide (Figure 1A). The central carbide was later shown to originate from the methyl function of the NifB SAM cofactor.^[6]^ This central carbide has further been identified via VtC XES on the L‐cluster, a FeMoco precursor, bound to the NifEN scaffold.^[7]^ Similar spectroscopic and structural studies revealed that FeVco contains an identical Fe_6_C core (Figure 1B).^[8]^ FeMoco and FeVco therefore share a similar structure, differing only by their heterometal and a carbonate replacing a belt sulfide in FeVco.[^8b^, ^9^] While the crystal structure of FeFe, the catalytic moiety of Fe nitrogenase, has yet to be reported, Mössbauer and EXAFS analyses suggested that FeFeco is structurally identical to FeMoco, with the only difference between these two cofactors being an iron atom replacing the molybdenum in FeFeco, which would therefore have a [8Fe:9S:C:homocitrate] composition.^[10]^ However, these analyses were performed before the identification of the carbide in FeMoco and FeVco. While the role of the carbide in the cofactors of nitrogenases remains unclear, crystal structures of CO‐ bound MoFe and VFe as well as recent ENDOR studies on MoFe showed that the Fe_6_C core conserves its native structure in the CO inhibited states.^[11]^ As these states are reached under turnover condition, these studies imply a stabilizing function for the carbide. Meanwhile, electrochemical measurements on carbyne‐ligated complexes suggested that the carbide renders the cofactor more reducing, thereby potentially more reactive towards N_2_.^[12]^

**Figure 1:**
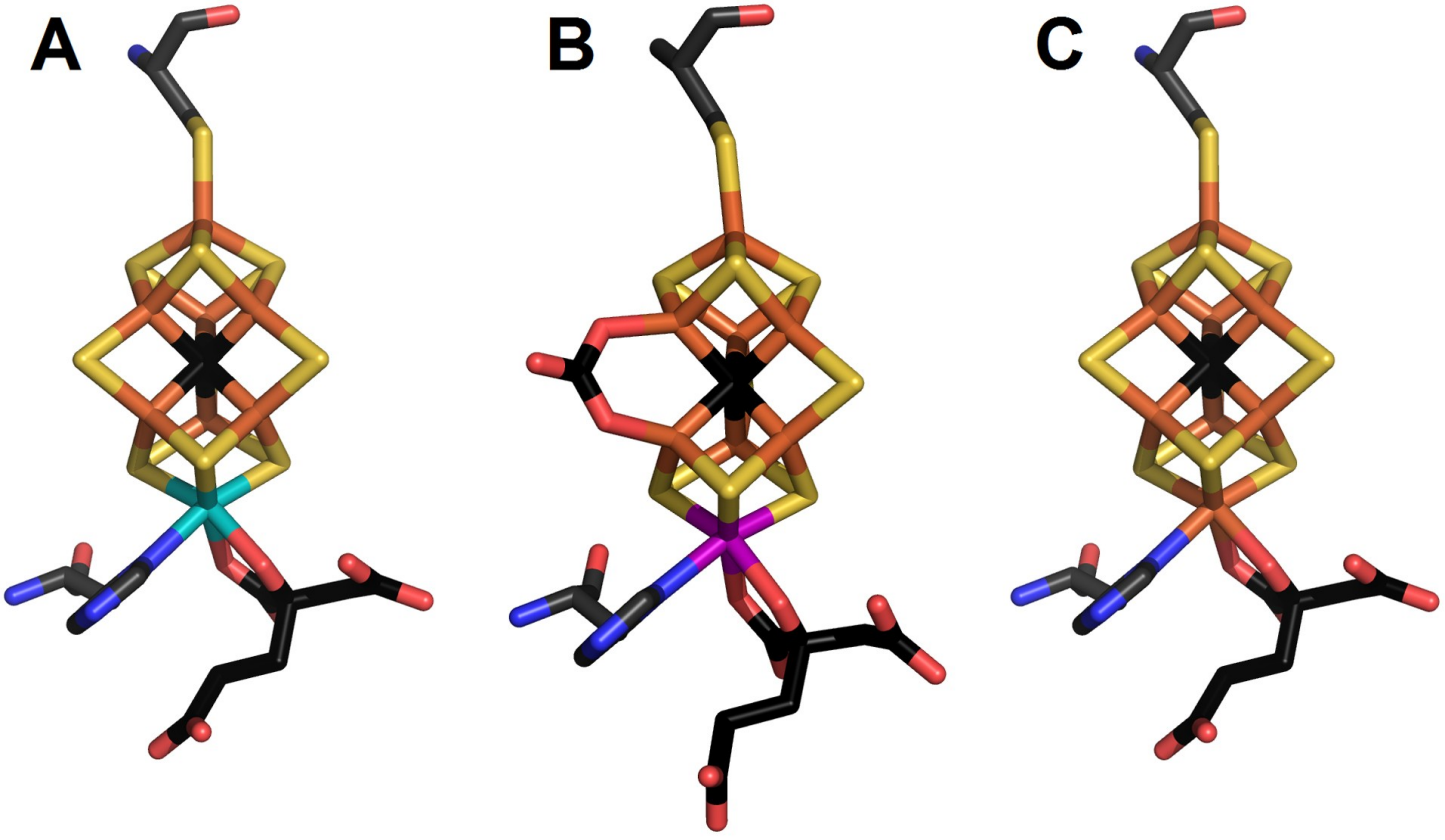
Structures of FeMoco in MoFe (PDB ID: 3U7Q)^[6b]^ (A) and FeVco in VFe (PDB ID: 5N6Y)^[8b]^ (B), and model of FeFeco in FeFe (C) proteins, coordinated by homocitrate and Cys and His side chains. Yellow: sulfur; orange: iron; black, carbon; red, oxygen; blue, nitrogen; teal, molybdenum; pink, vanadium.

To determine whether FeFeco also contains a carbide, making the Fe_6_C core a fundamental property of nitrogenase cofactors, we performed Fe Kβ VtC XES measurements on the FeFe protein of *Azotobacter vinelandii*.

VtC XES allows the observation of transitions between the dominantly ligand valence molecular orbitals (MOs) to the metal 1s core hole. After photoionization of a metal 1s electron, the electrons in ligand‐centered valence MOs relax to fill the generated 1s core hole, emitting fluorescent photons in the process. This results in the so called VtC XES region, which is comprised of a Kβ_2,5_ peak corresponding to ligand np→metal 1s transitions (≈7102 to 7112 eV), and the Kβ′′ feature corresponding to ligand ns→metal 1s transitions.^[13]^ The Kβ_2,5_ peak is modulated by the covalent interactions of the metal atom with the coordinated ligands, while the more localized nature of the ligand ns orbitals allows the Kβ′′ feature to serve as a more robust probe of ligand identity, particularly for monoatomic ligands. As such the Kβ′′ feature is an excellent spectroscopic marker for a light atom ligand, such as a carbide.^[14]^ We note that in contrast, it is not anticipated that a carbonate would be distinguishable from a bridging sulfide, as these ligands are expected to have overlapping contributions in the Kβ_2,5_ region and only very weak contributions to the Kβ′′ region, consistent with VFe and MoFe having identical VtC XES spectra.^[8a]^

The VtC XES spectrum of FeFe is shown in Figure 2, together with previously published data for the MoFe and VFe proteins. The FeFe VtC spectrum is nearly superimposable with that of MoFe and VFe, displaying a Kβ_2,5_ peak at 7108 eV, and a Kβ′′ peak at 7100 eV, the latter of which is attributed to the interstitial carbon in the cofactors. Hence, this result clearly evidences that FeFeco contains an Fe_6_C core similar to this in FeMoco and FeVco (Figure 1C).[^6a^, ^8a^]

**Figure 2:**
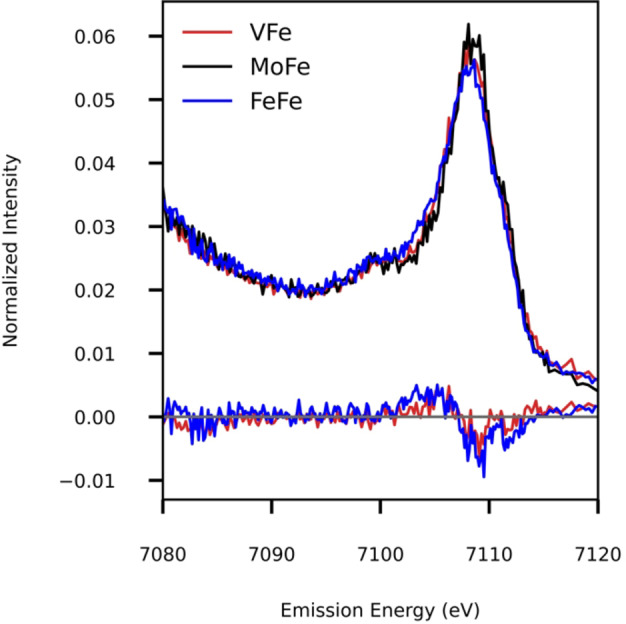
Fe Kβ VtC XES spectra of MoFe, VFe and FeFe proteins from *A. vinelandii*. Difference spectra relative to MoFe are shown below with (FeFe‐MoFe) in blue and (VFe‐MoFe) in red.

The difference spectra between FeFe and MoFe (Figure 2, blue) as well as that of VFe and MoFe (red) show no changes in the baselines or the Kβ′′ peak. However, subtle differences can be observed in the Kβ_2,5_ region between the three proteins, with the MoFe peak displaying a higher intensity compared to VFe and FeFe, and the FeFe peak being broader than MoFe and VFe. These differences could be due to the different heterometal in each cofactor or variations in bond lengths, while the Fe_6_C core remains the same.

Fe nitrogenase is often considered as the simplest nitrogenase isozyme, since its biosynthesis requires a smaller machinery than Mo and V nitrogenases.^[5b]^ No scaffold analogous to NifEN/VnfEN seems required for the maturation of its cofactor, suggesting FeFeco is analogous to NifB‐co with a bound homocitrate.^[4]^ This is consistent with Mössbauer analyses that established that FeFeco contains 8 iron atoms and displays a similar electronic structure to FeMoco and FeVco.^[10]^ However, contrary to FeMoco, FeFeco in the resting state is EPR‐silent, suggesting a [4Fe^II^4Fe^III^] content.^[15]^ This would resemble the electronic structure of NifB‐co as reported with Mössbauer and DFT experiments.^[16]^ The difference in metal content and overall charge may relate to the variations in reactivity between the different nitrogenases. Notably, while Fe nitrogenase is less efficient at reducing N_2_, it is a better catalyst for CO reduction, and was reported to reduce CO_2_ to CH_4_ in vivo.^[17]^ Our present VtC XES results on FeFe and our previous data on MoFe and VFe show that all nitrogenase cofactors contain a common Fe_6_C core.[^6a^, ^7^, ^8^] Our spectroscopy study on V nitrogenase preceded the X‐ray crystallography structural model, which revealed a carbonate ligand in FeV‐co undetected by VtC XES.[^8^, ^9^] Nevertheless, it is unlikely that FeFeco contains such a carbonate ligand, as the gene cluster encoding the machinery for Fe nitrogenase biosynthesis does not contain homologs to the *vnfP* genes suggested to be responsible for carbonate insertion in VFe.^[8b]^ The current study, however, clearly highlights that the Fe_6_C core is a central motif of all nitrogenase active sites and likely essential to the unique activity of these enigmatic cofactors.

## Conflict of interest

The authors declare no conflict of interest.

## Supporting information

As a service to our authors and readers, this journal provides supporting information supplied by the authors. Such materials are peer reviewed and may be re‐organized for online delivery, but are not copy‐edited or typeset. Technical support issues arising from supporting information (other than missing files) should be addressed to the authors.

Supporting Information

## Data Availability

The data that support the findings of this study are available from the corresponding author upon reasonable request.
