# Supplementary material for: An Fe6C Core in All Nitrogenase Cofactors
Source: Angew Chem Int Ed Engl. 2022 Sep 7;61(41):e202209190. doi: 10.1002/anie.202209190 (PMC9826452; doi:10.1002/anie.202209190)
Supplement: Supplementary file 1 — Supporting Information [file ANIE-61-0-s001.pdf]

Angewandte Chemie
Eine Zeitschrift der Gesellschaft Deutscher Chemiker

Supporting Information

An Fe_6C Core in All Nitrogenase Cofactors

L. Decamps, D. B. Rice, S. DeBeer**

Table of Contents

Experimental Procedures 2
Results and Discussion 2
References 3
Author Contributions 3

Experimental Procedures

Bacterial growth. *Azotobacter vinelandii* strain DJ2241 (generously provided by Prof. Dennis Dean, Virginia Polytechnic Institute and State University) was plated on Modified Burk medium agar plates containing no molybdenum or vanadium and supplemented with 10,5 mM ammonium chloride.[1] Following 3 days of incubation at 30°C, individual colonies were picked and incubated in liquid medium supplemented with 10,5 mM NH_4Cl at 30°C for 20 hours under shaking at 180 rpm. Then, 1 L medium (without ammonium) in a 5 L Erlenmeyer flask was inoculated with 10 mL of the preculture and further incubated at 30°C under shaking at 180 rpm until OD_{600} reached 1.8. Cells were harvested by centrifugation in an Avanti J-20 XP centrifuge at 6,500*g for 25' and stored at -80°C until lysis.

Protein isolation. All sample preparation steps were performed anaerobically in a Coy glovebox with a N_2 98%/H_2 2% atmosphere. Cell pellets were stirred in BugBuster MasterMix (Millipore), 5 mM sodium dithionite (4:1 v/w) for 1 h before being centrifuged at 45,000 rpm in an Optima LE-80K centrifuge (Beckman-Coulter) with a 70 Ti fixed-angle rotor (Beckman-Coulter). The soluble fraction was applied to a 20-mL Strep-Tactin column. The column was then washed with 80 mL buffer T [Tris 50 mM pH 7.4, NaCl 200 mM] and elution was performed with 40 mL buffer T + 2.5 mM desthiobiotin. ^{Str}FeFe protein was concentrated up to 26 mg/ml (corresponding to an effective Fe concentration of 3,5 mM) using 100 kDa MWCO concentrators (Millipore) and loaded in X-ray cells sealed with 38-micron Kapton tape before being frozen in liquid nitrogen.
Sample concentration was calculated using the DC Protein assay (Bio-Rad) following the Lowry method,[2] and purity was assessed via SDS-PAGE (Figure S1) using NuPAGE Bis-Tris 4-12% gels and NuPAGE MOPS SDS-running buffer (ThermoFisher).

XES Measurements and Analysis. XES data were collected at beamline ID26 of the European Synchrotron Radiation Facility (Grenoble, France). The ESRF storage ring was operating at 6 GeV, and the experiment was done with 200 mA current. Samples were kept at ~20 K with a displex He cryostat. The incident photon energy was set to 7800 eV using a Si(111) double crystal monochromator. The beam size at the sample was 0.05 (h) x 0.1 (v) mm. The incident energy was calibrated by setting the first inflection point of a Fe foil to 7111.2 eV. X-ray emission from the sample was measured using a Johann-type spectrometer equipped with an array of five spherically bent Ge (620) analyzer crystals aligned in the Rowland geometry in combination with an Avalanche Photodiode detector.
Radiation damage was assessed by performing short Fe Kβ high-energy resolution fluorescence detected (HERFD) X-ray absorption measurements (Figure S2) using a continuous scan mode from 7105 to 7150 eV with 0.10 eV step size and a total accumulation time of 10 s per scan. A final total dwell time per spot was determined to be 70 s by measuring successive scans on the same spot of the sample while attenuating the beam with a 80-mm aluminium foil.
VtC XES data were collected accordingly. The mainline XES was collected in sections of 7020 – 7035, 7035 – 7050, 7060 – 7065, and 7065 – 7080 eV (Figure S3). VtC spectra were collected in sections of 7080 – 7085 (step size 0.15 eV, dwell time 2 s/spot), 7085 – 7095 (step size 0.15 eV, dwell time 2 s/spot), 7095 – 7104 (step size 0.15 eV, dwell time 1.1 s/spot), 7104 – 7113 eV (step size 0.15 eV, dwell time 1.1 s/spot), and 7114 – 7130 eV (step size 0.3 eV, dwell time 1.1 s/spot). Spot-to-spot sample concentration inhomogeneity was accounted for by normalizing the spectra from each sample spot to its respective counts at the K$\beta_{1,3}$ maximum collected after the VtC. The emission spectra were referenced to Fe_2O_3 K$\beta_{1,3}$ maxima of 7060.6 eV and the K$\beta_{2,5}$ peak at 7107.2 eV. For comparison to previously published MoFe and VFe data,[3] the spectra were referenced to the Fe_2O_3 collected at each beamtime using the above values.
Data were processed using Python 3.8.8 using the h5py and numpy packages.[4]

Results and Discussion

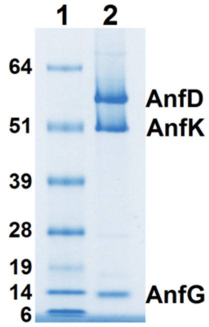

Figure S1: SDS-PAGE analysis of the FeFe sample analysed in the present study. Lane 1: SeeBlue Prestained marker, lane 2: FeFe (100 µg). Molecular weights are shown in kDa.

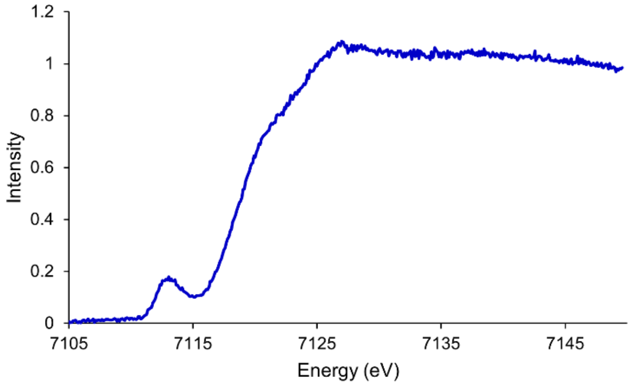

Figure S2: Fe Kβ HERFD XAS spectrum of the FeFe protein.

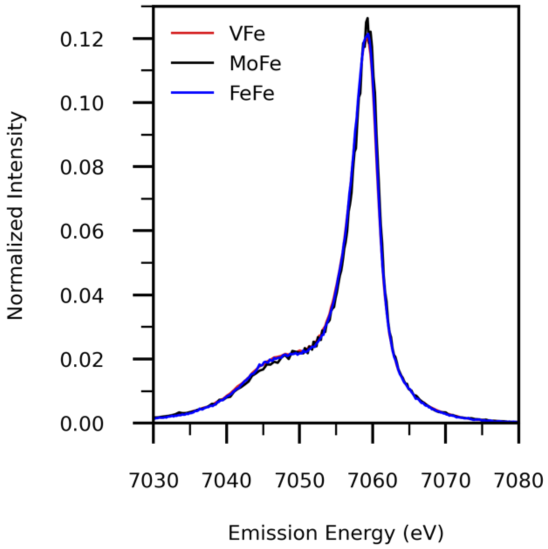

Figure S3: Kβ mainline of the FeFe protein along with the MoFe and FeFe data collected previously. Spectra are normalized to a total area of 1.

References

[1] a) A. Pérez-González, E. Jimenez-Vicente, J. Gies-Elterlein, A. Salinero-Lanzarote, Z.-Y. Yang, O. Einsle, C. Seefeldt Lance, R. Dean Dennis, L. Johnson Michael David, *mBio* **2021**, *12*, e01568-01521; b) A. Perez-Gonzalez, E. Jimenez-Vicente, A. Salinero-Lanzarote, D. F. Harris, L. C. Seefeldt, D. R. Dean, *Mol Microbiol* **2022**; c) G. W. Strandberg, P. W. Wilson, *Can J Microbiol* **1968**, *14*, 25-31.

[2] O. H. Lowry, N. J. Rosebrough, A. L. Farr, R. J. Randall, *Journal of Biological Chemistry* **1951**, *193*, 265-275.

[3] a) K. M. Lancaster, M. Roemelt, P. Ettenhuber, Y. L. Hu, M. W. Ribbe, F. Neese, U. Bergmann, S. DeBeer, *Science* **2011**, *334*, 974-977; b) J. A. Rees, R. Bjornsson, J. Schlesier, D. Sippel, O. Einsle, S. DeBeer, *Angew Chem Int Edit* **2015**, *54*, 13249-13252.

[4] C. R. Harris, K. J. Millman, S. J. van der Walt, R. Gommers, P. Virtanen, D. Cournapeau, E. Wieser, J. Taylor, S. Berg, N. J. Smith, R. Kern, M. Picus, S. Hoyer, M. H. van Kerkwijk, M. Brett, A. Haldane, J. F. Del Rio, M. Wiebe, P. Peterson, P. Gerard-Marchant, K. Sheppard, T. Reddy, W. Weckesser, H. Abbasi, C. Gohlke, T. E. Oliphant, *Nature* **2020**, *585*, 357-362.

Author Contributions

L.D. and S.D. designed the project. S.D. acquired funding. L.D. and D.R. carried out the measurements. D.R. analysed the data. All authors discussed and interpreted the results. L.D. wrote the manuscript and D.R. and S.D. participated in manuscript preparation and draft revision.
